# Morning priming exercises for explosive performance: time course effects of two-way ballistic and strength-based protocols

**DOI:** 10.5114/biolsport.2026.158671

**Published:** 2026-01-23

**Authors:** Yiheng Zeng, Tao Wu, Junlei Lin, Wei Li, Paul B. Gastin, Olivier Girard

**Affiliations:** 1 School of Strength and Conditioning Training, Beijing Sport University, Beijing, China;; 2 Force-velocity Laboratory, Beijing Sport University, Beijing, China; 3 Department of Sport and Exercise Science, La Trobe University, Melbourne, Australia; 4 School of Human Sciences (Exercise and Sports Science), The University of Western Australia, Perth, Western Australia, Australia

**Keywords:** Two-way ballistic, Concentric-assisted, Standing broad jump, Delayed potentiation effect, Countermovement jump

## Abstract

To examine the effects of low- and high-intensity two-way ballistic (TWB) priming exercise versus high-intensity traditional strength exercise (TSE) on explosive performance at 6 h and 24 h post-intervention. Twenty-eight well-trained male athletes completed four randomized morning sessions (08:00–11:00) in separate weeks: 30% 1RM TWB, 80% 1RM TWB, 80% 1RM TSE, and control. Each priming protocol comprised three sets of six repetitions with 4-min rest intervals. Explosive performance was assessed via countermovement jump (CMJ), five consecutive CMJs, and standing broad jump, measured pre-intervention, and at 6 h and 24 h post. After 6 h, all exercise conditions (excluding control) significantly improved CMJ height (30% 1RM TWB: 6.4 ± 5.0%, d = 1.31; 80% 1RM TWB: 7.4 ± 3.1%, d = 2.49; 80% 1RM TSE: 3.5 ± 4.2%, d = 0.83; all p < 0.01) and relative peak power (3.5 ± 1.8%, d = 1.87; 4.8 ± 1.8%, d = 2.83; 3.2 ± 2.1%, d = 1.53; all p < 0.01). Across conditions, average height across consecutive CMJs increased, with improvements of 6.7 ± 3.5% (d = 1.96), 7.6 ± 6.6% (d = 1.18), and 4.2 ± 2.7% (d = 1.53) for 30% 1RM TWB, 80% 1RM TWB, and 80% 1RM TSE, respectively (all p < 0.01). Similar patterns were observed for reactive strength index-modified (12.4 ± 12.1%, d = 0.95; 9.4 ± 11.7%, d = 0.79; 9.4 ± 14.1%, d = 0.71; all p < 0.01), and standing broad jump distance (1.9 ± 1.2%, d = 1.52; 2.4 ± 1.6%, d = 1.52; 1.1 ± 1.5%, d = 0.72; all p < 0.01). At 24 h, performance gains were largely diminished, with the 30% 1RM TWB condition returning to baseline (all p > 0.05). Both high-intensity protocols (TWB and TSE) maintained improvements, with the TWB protocol yielding the most consistent benefits, particularly in CMJ and CCMJ measures. Morning TWB priming at both low and high intensities enhances explosive performance within 6 h, making it a viable delayed potentiation strategy for sports with afternoon or evening competition. High-intensity TWB sustains benefits up to 24 h, indicating greater suitability for high-intensity or competitive settings requiring prolonged performance readiness.

## INTRODUCTION

The delayed potentiation effect (DPE) is characterized by a short-term enhancement in neuromuscular performance following sufficiently intense resistance exercise, with effects typically lasting 2–48 hours [1, 2]. Unlike post-activation performance enhancement, which occurs within 4–20 minutes post-conditioning activity [3], DPE develops more gradually and persists longer, and is thought to arise from a complex interplay of integrative mechanisms [4–6]. From a hormonal perspective, DPE may be supported by attenuated diurnal declines in free testosterone and maintenance of the testosterone-to-cortisol ratio, helping preserve an anabolic internal environment and readiness to perform [4]. However, endocrine responses are often highly variable and may fall below thresholds of biological relevance, suggesting that hormonal changes may facilitate rather than directly drive DPE [4]. From a neuromuscular perspective, priming exercise can transiently enhance corticospinal excitability and motor unit recruitment, improving neural drive and coordination [5]. Mechanically, increases in muscle and tendon stiffness during recovery – potentially linked to low-level inflammation or modified coordination patterns–may improve force transmission and fascicletendon behavior, thereby enhancing the rate of force development and power output [5]. These effects are typically task-specific, reflecting the retention of neuromotor patterns aligned with the priming activity. Finally, goal-priming and expectancy effects may amplify these physiological responses through increased motivation, attention, and perceived readiness. Together, these endocrine, neuromechanical, and psychological factors contribute to a temporary enhancement of performance capacities – such as jumping, sprinting, or power output – detectable 6–24 hours after an appropriately designed priming exercise stimulus [6]. This phenomenon holds significant implications for athletes, as strategically timed resistance exercise before competition may boost subsequent performance.

Considering the optimal timing of priming exercise, most studies have applied morning-based protocols performed ~6 h before competition [7–9]. Maintaining DPE over longer periods is more challenging than with a 6-h priming protocol and depends on factors such as priming method, intensity, and training volume [5, 10, 11, 12–14]. High-intensity resistance exercise has been shown to significantly enhance neuromuscular function and athletic performance [15]. For instance, Cook et al. [2]reported significant improvements in squat strength, countermovement jump (CMJ), and sprint performance 6 h after a 3-repetition maximum (RM) squat protocol. On the other hand, no studies have confirmed that low-intensity resistance exercise (< 30% 1RM) induces DPE, except for ballistic exercise (BE), where low intensities (e.g., 30% 1RM) have been shown to improve CMJ height [16]. Saez et al. [17] further demonstrated that while 30% 1RM half-squats failed to enhance performance 6 h post-exercise, loaded squat jumps at the same intensity significantly increased CMJ height. Collectively, these findings suggest that velocity-oriented priming exercise at low intensity may still elicit DPE.

**FIG. 1:**
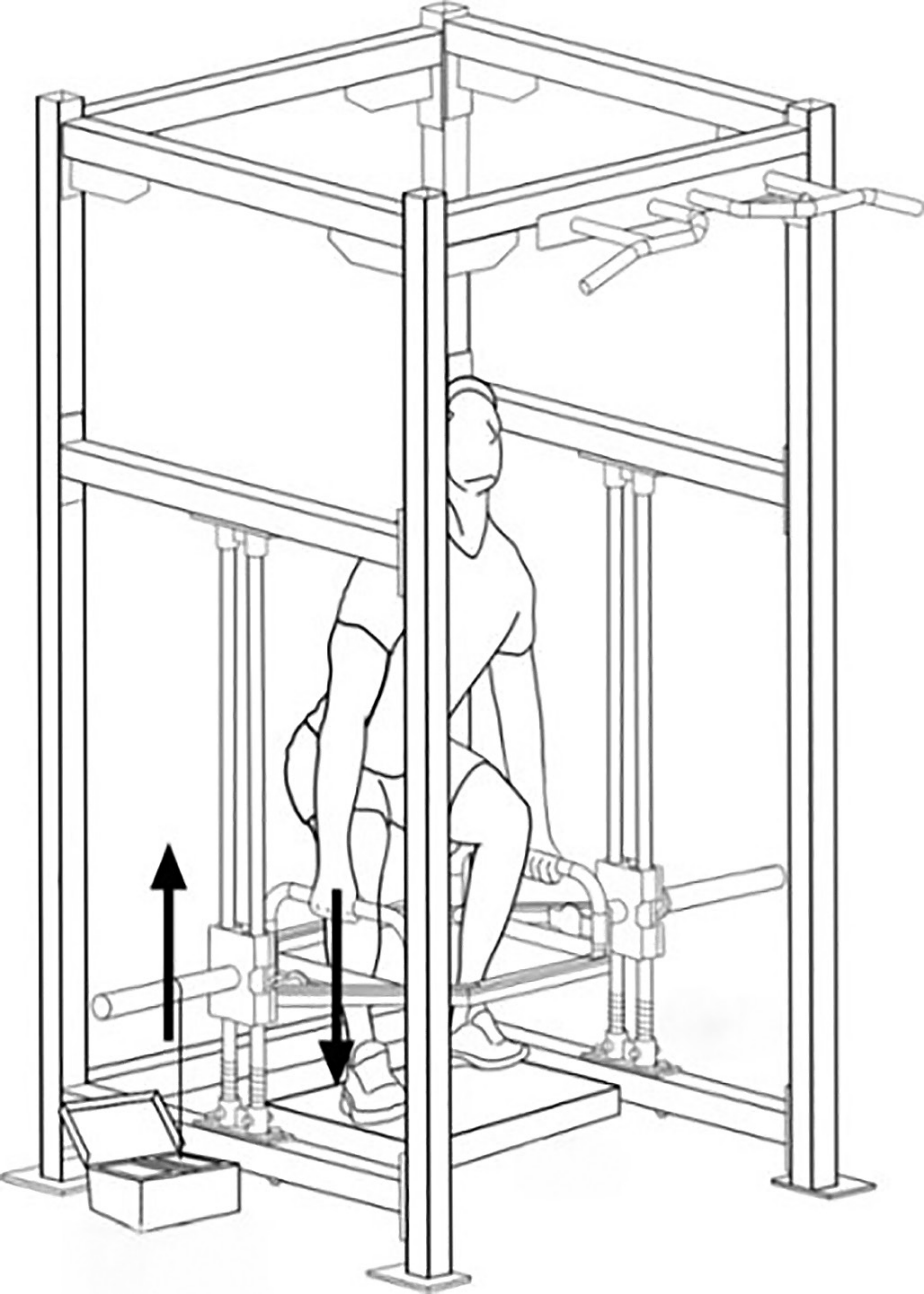
Trap bar machine

BE is traditionally used to develop explosive performance by emphasizing the concentric phase performed with maximal intended velocity [18]. However, its focus on unidirectional acceleration and single-effort execution limits movement continuity, making it less suitable for sports requiring continuous, bidirectional actions. To overcome this limitation, we developed a two-way ballistic (TWB) training approach for multi-joint exercises using a custom-designed trap bar device (Figure 1). In this set-up, the upward phase is assisted by a spring rebound at the bottom, enabling maximal velocity to be achieved in both movement directions.

This study aimed to assess DPE on explosive performance at 6 h and 24 h post-intervention, comparing low- and high-intensity TWB priming exercises with high-intensity traditional strength exercise (TSE) using a customized trap bar machine. We hypothesized that both low- or high-intensity TWB would elicit greater DPE relative to TSE, supporting TWB as a promising strategy to boost explosive performance.

## MATERIALS AND METHODS

### Participants

Twenty-eight well-trained male college sprinters participated (age: 19.9 ± 1.4 years; height: 177.8 ± 5.0 cm; body mass: 72.6 ± 6.6 kg; trap bar deadlift 1RM: 2.08 ± 0.22 × body mass; 100-m personal best time: 11.11 ± 0.39 s). Participants were categorized as “Highly trained” (Tier 3) based on established criteria [19]. Inclusion criteria required at least two years of sprint-specific training, a trap bar deadlift 1RM > 1.5 × body mass, regular training (> 2 sessions/ week, ≥ 90 min/session), and no history of major diseases or serious sports injuries. Participants were instructed to avoid strenuous exercise for 72 hours before testing. Although a prior power analysis was initially conducted, the effect size was based on previous literature and may not fully represent the present study design. Thus, a post hoc power analysis (1-β) was performed in G*Power, using the converted partial η^2^ from the two-way repeated-measures AVOVA for CMJ height (ηp^2^ = 0.268), which corresponded to an effect size f = 0.605. Consequently, the achieved power was 1–β = 0.99, indicating that the study was adequately powered to detect the observed interaction effect. The study was approved by the Sports Science Experiment Ethics Committee of Beijing Sport University Ethics Review Board for Human Participants (no. 2025521H) and complied with the principles of the Declaration of Helsinki. Written informed consent was obtained from all participants.

### Experimental design

A randomized crossover design was used to compare DPE of three interventions: 30% 1RM TWB, 80% 1RM TWB and 80% 1RM TSE. Each involved three sets of six repetitions with 4-min rest intervals. A non-intervention control condition was included to account for diurnal fluctuations in performance [20]. Performance was assessed at 6 and 24 h post-intervention. Force-generating capability was measured via CMJ height and relative peak power, average height from five consecutive CMJs (CCMJ), reactive strength index modified (RSImod), and standing broad jump (SBJ) distance. Each test was performed three times with 2–3 minutes of rest between trials.

### Procedures

**FIG. 2:**
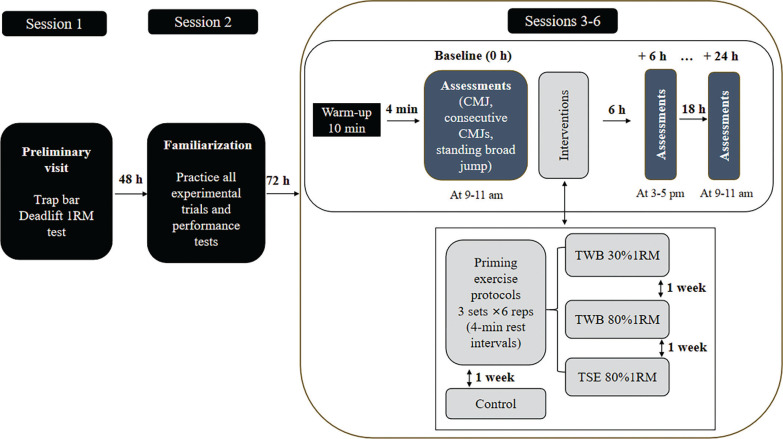
The study design

Participants first completed a preliminary visit to determine their trap bar deadlift 1RM, following the protocol described by Hagerupsen et al. [21]. Specifically, participants completed four warm-up sets consisting of 8–10 repetitions at 50%, 6 repetitions at 70%, 3 repetitions at 80%, and 1 repetition at 90% of their estimated 1RM. They were then permitted as many attempts as necessary to achieve a successful 1RM, though they were encouraged to do so within five attempts. The load was increased by 2.5–10 kg for each successful attempt, with each attempt consisting of one repetition and separated by at least three minutes of passive rest. The heaviest load successfully lifted for one repetition was reported as the participant’s 1RM. After 48 h, they attended a single familiarization session to practice all experimental procedures, including TWB, TSE, and performance measurements. These were executed at 75% of maximal effort, with 1–2 min of rest between trials. The following week, participants began the first of four main experimental trials, each separated by one week. Every session started between 08:00 and 11:00 and began with a standardized 10-min warm-up (5 min jogging and 5 min self-selected dynamic stretching), followed by 4 min of passive rest. Baseline (pre-test) assessments were then performed in a randomized order, with each test executed three times with 2-min rest intervals. Performance measures included CMJ height and relative peak power, CCMJ average height, RSImod, and SBJ distance. The same warm-up and testing procedures were conducted at 6 h post-intervention (14:00–17:00 on the same day) and again at 24 h (08:00–11:00 the next day). Each participant was tested at consistent intervals, with time-of-day variability kept within ± 1 h. All tests were conducted indoors in a gymnasium (Figure 2).

### Two-way ballistic priming exercise

A customized, spring-assisted device was used to provide concentric assistance during multi-joint dynamic movements. The trap bar was guided along a fixed vertical trajectory and incorporated a spring mechanism at the bottom, allowing it to rebound and increase upward velocity. This setup enabled participants to complete the movement ‘more explosively’ under the same load, thereby facilitating concentric-assisted training.

**FIG. 3:**
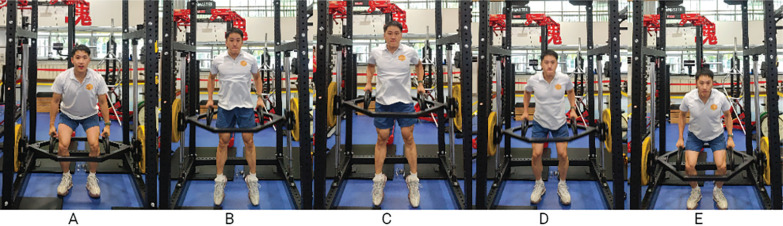
Phases of the two-way ballistic (TWB) movement: start position (A); upward concentric phase (B); highest point of the amortization phase (C); downward concentric phase (D); lowest point of the amortization phase (E).

For TWB training, participant stood upright holding the trap bar. They first pushed the bar downward with maximal effort. Upon spring contact, the bar rebounded upward, and participants pulled upward with full strength. At the apex, they pushed downward again with maximal effort, repeating this cycle for three sets of six continuous repetitions (Figure 3).

### Traditional strength priming exercise

Participants performed trap-bar deadlifts at 80% 1RM, completing three sets of six repetitions. For each set, participants stood centrally within the trap bar with feet approximately hip-width apart, toes pointing slightly outward, heels flat, and hands on the neutral handles. The starting position required hips and knees flexed so that the bar sat just below the patella, with a neutral lumbar spine, retracted scapulae, chest up, and eyes fixed on a spot 2–3 m ahead. At the investigator’s command, participants initiated the lift with a controlled hinge and then produced maximal concentric effort to full hip and knee extension without lumbar hyperextension. The bar was then lowered under control to the floor after each repetition. Repetition tempo was self-selected, but participants were instructed to perform the concentric phase as fast as possible (maximal intended velocity) and to complete the descent in a controlled manner.

### Countermovement jump

Participants began each CMJ from a standing position with knees and hips fully extended, feet approximately shoulder-width apart and slightly externally rotated. Hands were placed on the hips to eliminate arm-swing contributions. Participants performed a fast countermovement descent to a self-selected depth and then jumped as high as possible through coordinated knee and hip extension. All jumps were performed on a force platform (KWYP-FPW6035, Kun-Wei) sampling at 1000 Hz, with a 5 N threshold used to identify take-off and landing events [22]. Vertical jump height was calculated using flight time (i.e., the period between takeoff and subsequent ground contact), applying the equation: jump height = (flight time)^2^ × g/8. Data showed excellent intrasession test-retest reliability (ICC_CMJ_ = 0.98).

### Consecutive countermovement jumps

Participants started from the same standing CMJ position (see above), with hands on the hips to eliminate arm swing, and performed five consecutive maximal-effort jumps without pauses, initiating each jump immediately upon ground contact. Data were sampled at 1000 Hz (KWYP-FPW6035, KunWei). Average vertical jump height across the five jumps and RSImod (RSImod = average CCMJ height / average ground contact time) were calculated using KunWei software (version 2.0; KunWei Corporation). Vertical jump height was derived from flight time using the equation: jump height = (flight time)^2^ × g/8. Data showed moderate intrasession test-retest reliability (ICC_CCMJ_ = 0.82).

### Standing broad jump

Participants stood in a comfortable bilateral stance with their feet hip-width apart, ensuring that their toes remained behind the starting line. From this position, they performed a self-selected counter-movement and then maximally propelled themselves forward by rapidly extending the hips, knees and ankles while swinging their arms naturally. Participants were instructed to land on both feet simultaneously, maintain balance, and avoid taking any additional steps. The horizontal distance from the take-off line to the nearest point of contact on landing (typically the heels) was measured to the nearest 0.5 cm using a steel measuring tape [23]. Data showed moderate intrasession test-retest reliability (ICC_SBJ_ = 0.77).

### Statistical analysis

Descriptive data are presented as mean (SD). Data normality was assessed using the Shapiro-Wilk test, followed by variance homogeneity evaluation. All variables were analyzed with a two-way repeated measures ANOVA (time [0, 6 and 24 h] × condition [30% 1RM TWB, 80% 1RM TWB, 80% 1RM TSE, and control]). Post hoc comparisons were Bonferroni-adjusted. Cohen’s *d* was calculated to quantify effect sizes, with d < 0.2 = *trivial*, 0.2 ≤ d < 0.5 = *small*, 0.5 ≤ d < 0.8 = *moderate*, and d ≥ 0.8 = *large* [24]. Given the repeated-measures crossover design, effect sizes were calculated as Cohen’s d for paired samples, using the mean of the individual change scores (post–pre) divided by the standard deviation of those change scores. This approach accounts for within-subject dependency and does not rely on pooled standard deviations.

The smallest worthwhile change (SWC) was defined as 0.2 × the baseline standard deviation. Based on this threshold, individuals were classified as *responders* (changes > +SWC), *non-responders* (changes < –SWC), or *neutral responders* (changes within ± SWC). Statistical analyses were performed using SPSS statistical software (SPSS 25.0, IBM, New York, USA). The significance level was set at *p* < 0.05.

Intra-day reliability was calculated using both relative (intra-class correlation coefficient; ICC) and absolute (coefficient of variation; CV%) reliability. CV was calculated from the TE and expressed as a percentage. The thresholds used to interpret ICC values were ≤ 0.49 = *poor*, 0.50–0.74 = *moderate*, 0.75–0.89 = *good*, ≥ 0.90 = *excellent* [25]. The CV% was interpreted as > 15% = *poor*; 10–15% = *moderate*; 5–10% = *good*; < 5% = *excellent*. Overall reliability was interpreted by combining both the ICC and CV% classifications: ICC > 0.9 and CV% < 5% = *excellent*; ICC 0.75–0.9 and CV% < 10% = *good*; ICC < 0.75 and CV% > 10% = *moderate*; ICC < 0.75 and CV% < 10% = *poor* [26]. The 90% confidence intervals for all reliability estimates were also included. Typical error (TE) was compared to the SWC. SWC was calculated by multiplying the between-subject SD by 0.2 (SWC_0.2_) for a *small* effect or 0.5 (SWC_0.5_) for a *moderate* effect. If the TE was lower than the SWC, the test variable was rated ‘good’; if equal to the SWC, it was rated ‘OK’, and if higher than the SWC, it was rated ‘poor’.

## RESULTS

**FIG. 4:**
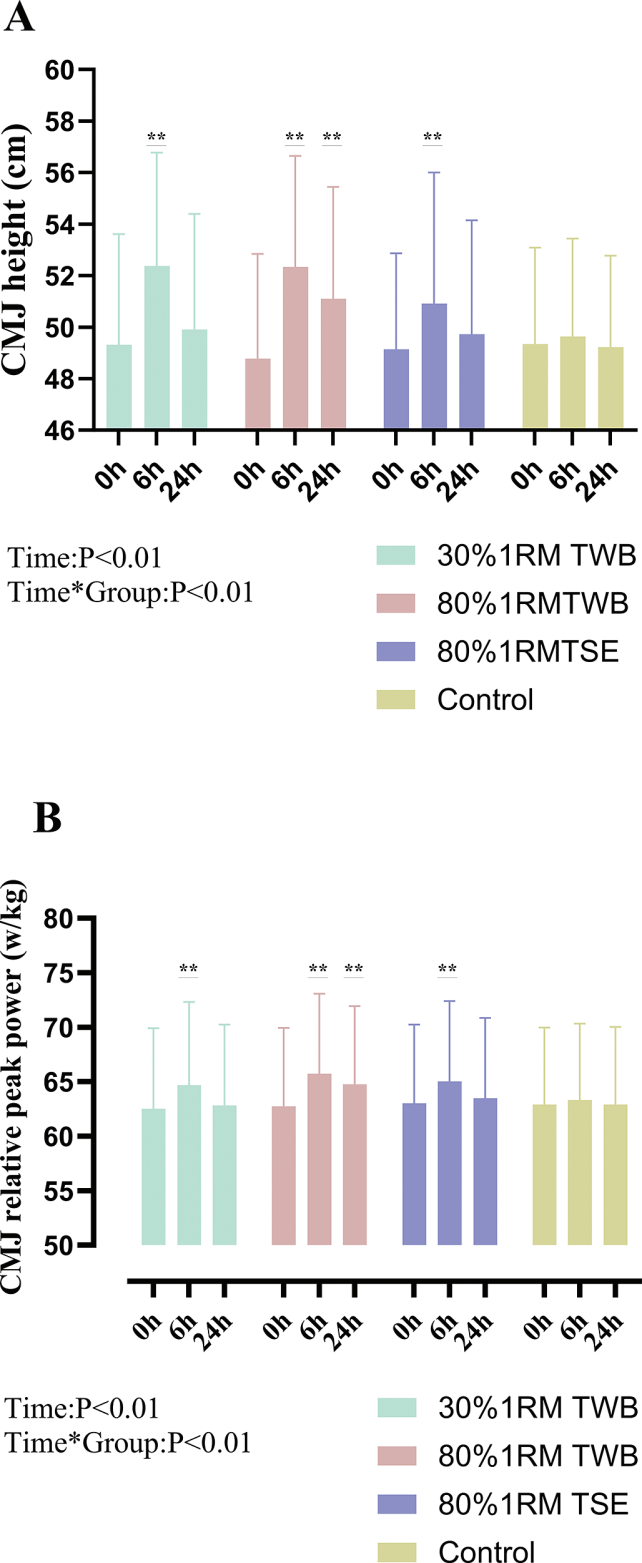
Countermovement jump (CMJ) height (A) and relative peak power (B) at baseline (0 h) and 6 and 24 hours after a morning priming exercise protocol. Participants performed the four following protocols one week apart: 30% 1RM two-way ballistic (TWB), 80% 1RM TWB, 80% 1RM traditional strength exercise (TSE), and control. ** (P < 0.01) denotes a significant difference from baseline.

**FIG. 5:**
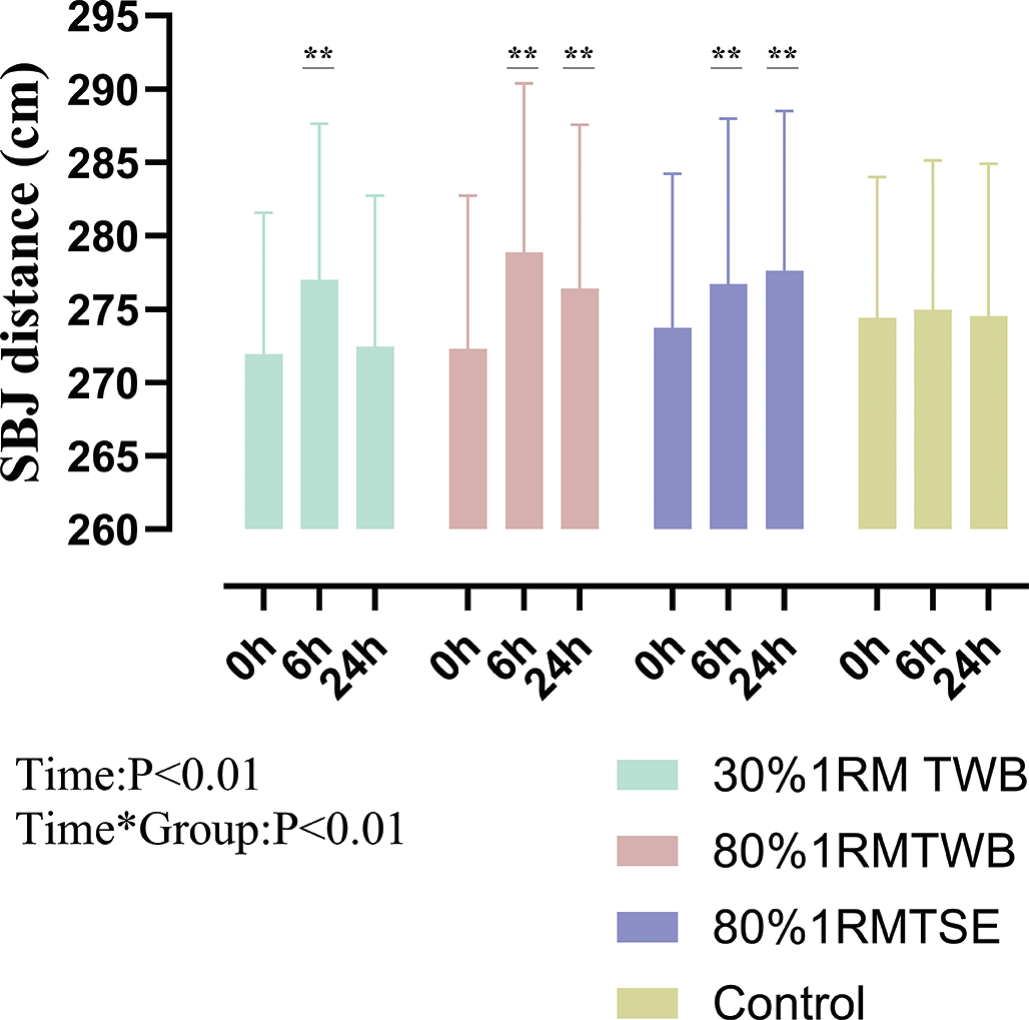
Standing broad jump (SBJ) distance at baseline (0 h) and 6 and 24 hours after a morning priming exercise protocol. Participants performed the four following protocols one week apart: 30% 1RM two-way ballistic (TWB), 80% 1RM TWB, 80% 1RM traditional strength exercise (TSE), and control. ** (P < 0.01) denote significant difference from baseline.

**FIG. 6:**
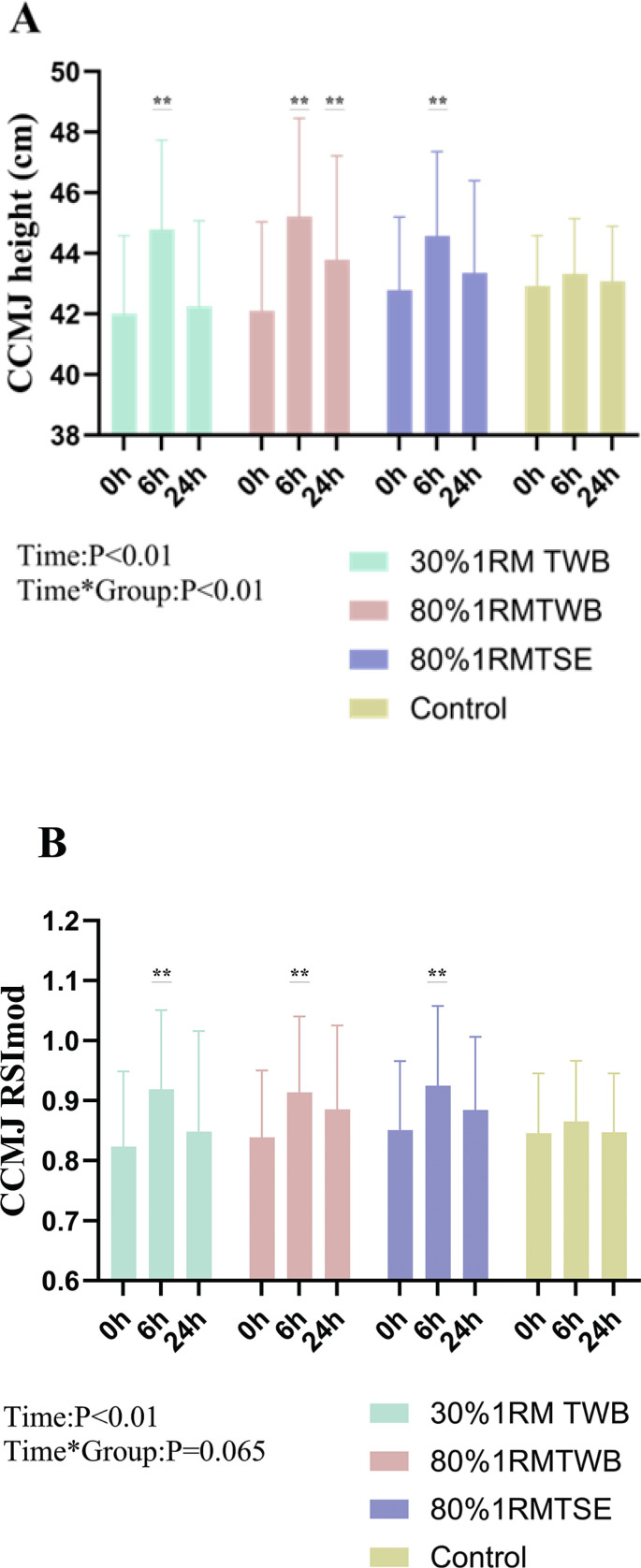
Continuous Countermovement jumps (CCMJ) average height (A) and CCMJ RSImod (B) at baseline (0 h) and 6 and 24 hours after a morning priming exercise protocol. Participants performed the four following protocols one week apart: 30% 1RM two-way ballistic (TWB), 80% 1RM TWB, 80% 1RM traditional strength exercise (TSE), and control. ** (P < 0.01) denote significant difference from baseline.

**FIG. 7:**
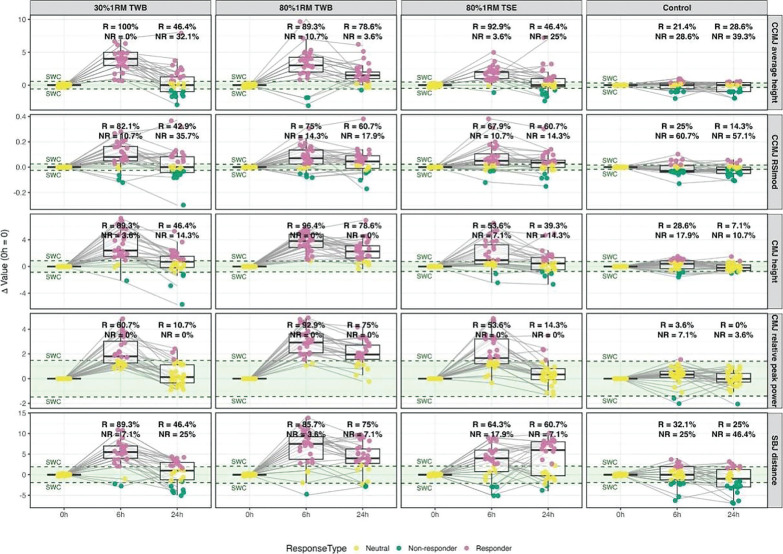
Individual responses graph at baseline (0 h) and 6 h and 24 hours after different morning priming exercise protocols: 30% 1RM two-way ballistic exercise (TWB), 80% 1RM TWB, 80% 1RM traditional strength exercise (TSE), and control. ‘R’ represents percentage of responders, and ‘NR’ represents the percentage of non-responders. SWC = 0.2 × baseline ± SD * (P < 0.05) denotes significant difference between 6 h and baseline; # (P < 0.05) denotes significant difference between 24 h and baseline; & (P < 0.05) denotes significant difference between 6 h and 24 h.

Two-way ANOVA revealed significant condition × time interactions for CMJ height (ηp2 = 0.268, *p* < 0.01), CMJ relative peak power (ηp^2^ = 0.367, *p* < 0.01), CCMJ average height (ηp^2^ = 0.231, *p* < 0.01), and SBJ distance (ηp^2^ = 0.234, *p* < 0.01), but not for CCMJ RSImod (ηp^2^ = 0.053, *p* = 0.065). No significant changes were observed in the control condition at 6 or 24 h relative to baseline. In the 30% 1RM TWB condition, no significant differences were found at 24 h in reference to baseline for any variables. Group-specific results are presented in Table 1 and Figures 4–7.

**TABLE 1:** Participants performed the four following protocols one week apart: 30% 1RM two-way ballistic (TWB), 80% 1RM TWB, 80% 1RM traditional strength exercise (TSE), and control.

Indicator	Intervention	Baseline	Post		Effect Sizes (95%CI)		Group × time (*p*)

			6 h	24 h	6 h vs Baseline	24 h vs Baseline	
CMJ height	30%TWB	49.3 ± 4.3	52.4 ± 4.4**	49.9 ± 4.5	1.31 (2.16, 3.97)	0.30 (-0.18, 1.38)	< 0.01
	80%TWB	48.8 ± 4.1	52.3 ± 4.3**	51.1 ± 4.3**	2.49 (3.01, 4.12)	1.45 (1.70, 2.95)	
	80%TSE	49.2 ± 3.7	50.9 ± 5.1	49.7 ± 4.4	0.83 (0.95, 2.60)	0.36 (-0.55, 1.22)	
	Control	49.3 ± 3.7	49.6 ± 3.8	49.2 ± 3.6	0.34 (-0.04, 0.62)	-0.20 (-0.35, -0.11)	

CMJ peak power · kg−1	30%TWB	62.51 ± 7.41	64.68 ± 7.64**	62.83 ± 7.42	1.87 (1.72, 2.62)	0.36 (-0.02, 0.67)	< 0.01
	80%TWB	62.74 ± 7.20	65.73 ± 7.34**	64.76 ± 7.18**	2.83 (2.58, 3.40)	2.01 (1.63, 2.41)	
	80%TSE	63.03 ± 7.20	65.03 ± 7.36**	63.49 ± 7.37	1.53 (1.49, 2.51)	0.43 (0.05, 0.86)	
	Control	62.91 ± 7.05	63.31 ± 7.02	62.90 ± 7.14	0.46 (0.07, 0.74)	-0.02 (-0.28, 0.05)	

SBJ distance	30%TWB	271.9 ± 9.6	277.0 ± 10.6**	272.5 ± 10.3	1.52 (3.77, 6.37)	0.18 (-0.60, 1.67)	< 0.01
	80%TWB	272.3 ± 10.4	278.9 ± 11.5**	276.4 ± 11.2**	1.52 (4.92, 8.29)	1.11 (2.69, 5.60)	
	80%TSE	273.7 ± 10.5	276.7 ± 11.3**	277.6 ± 10.9**	0.72 (1.39, 4.61)	0.91 (2.23, 5.55)	
	Control	274.4 ± 9.6	275.0 ± 10.2	274.5 ± 10.4	0.25 (-0.47, 2.25)	0.02 (-1.33, 1.47)	

CCMJ average height	30%TWB	42.0 ± 2.6	44.8 ± 3.0**	42.3 ± 2.8	1.96 (1.32, 2.59)	0.20 (-0.17, 0.58)	< 0.01
	80%TWB	42.1 ± 2.9	45.2 ± 3.2**	43.79 ± 3.4**	1.18 (2.08, 4.13)	1.07 (1.07, 2.29)	
	80%TSE	42.8 ± 2.4	44.6 ± 2.8**	43.4 ± 3.0	1.53 (1.33, 2.24)	0.32 (-0.12, 1.27)	
	Control	42.9 ± 1.7	43.3 ± 1.8	43.1 ± 1.8	0.35 (-0.05, 0.83)	0.12 (-0.34, 0.62)	

CCMJ RSImod	30%TWB	0.82 ± 0.13	0.92 ± 0.13**	0.85 ± 0.17	0.95 (0.06, 0.13)	0.20 (-0.02, 0.07)	0.065
	80%TWB	0.84 ± 0.11	0.91 ± 0.13**	0.89 ± 0.14	0.79 (0.04, 0.11)	0.47 (0.01, 0.08)	
	80%TSE	0.85 ± 0.11	0.93 ± 0.13**	0.88 ± 0.12	0.71 (0.03, 0.11)	0.40 (0.00, 0.07)	
	Control	0.85 ± 0.10	0.87 ± 0.10	0.85 ± 0.10	0.25 (-0.01, 0.05)	0.02 (-0.03, 0.03)	

Countermovement jump (CMJ) height and CMJ relative peak power, standing broad jump (SBJ) distance, continuous countermovement jumps (CCMJ) average height and CCMJ reactive strength index-modified (RSImod) were assessed at baseline and 6 and 24 hours post-intervention.

** (P < 0.01) denote significant difference from baseline.

### Intra-day reliability and sensitivity

The mean ± standard deviation for all variables across three trials are shown in table 2. Corresponding ICC, CV%, TE, and SWC values employed to determine intra-day and test sensitivity are presented in table 3. CMJ height (ICC = 0.98, CV = 0.94%), CMJ relative peak power (ICC = 0.97, CV = 1.05%), and CCMJ RSImod (ICC = 0.92, CV = 7.2%) had *excellent* overall intra-test reliability and were able to detect the SWC_0.2_. SBJ distance (ICC = 0.77, CV = 1.74%) and CCMJ average height (ICC = 0.82, CV = 3.85%) had moderate overall intra-test reliability and were able to detect the SWC_0.5_.

**TABLE 2:** Intra-day reliability and sensitivity of variables collected from three trials.

Variable	Intervention	Trial 1			Trial 2			Trial 3		

		0 h	6 h	24 h	0 h	6 h	24 h	0 h	6 h	24 h
CMJ height (cm)	30%TWB	49.1 ± 4.3	52.2 ± 4.3	49.7 ± 4.4	49.4 ± 4.3	52.5 ± 4.4	50.0 ± 4.5	49.4 ± 4.4	52.5 ± 4.5	50.1 ± 4.6
	80%TWB	48.5 ± 4.1	52.2 ± 4.3	50.9 ± 4.3	48.9 ± 4.0	52.4 ± 4.3	51.1 ± 4.4	48.9 ± 4.1	52.5 ± 4.4	51.3 ± 4.4
	80%TSE	49.2 ± 3.8	51.0 ± 5.1	49.8 ± 4.4	49.4 ± 3.9	51.2 ± 5.0	50.0 ± 4.5	49.6 ± 3.8	51.3 ± 5.1	50.1 ± 4.4
	Control	49.2 ± 3.7	49.5 ± 3.7	49.1 ± 3.7	49.6 ± 3.7	49.8 ± 3.7	49.5 ± 3.6	49.4 ± 3.9	49.7 ± 4.0	49.3 ± 3.6

CMJ relative peak power (W/kg−1)	30%TWB	62.39 ± 7.42	64.37 ± 7.61	62.64 ± 7.25	62.67 ± 7.43	64.67 ± 7.67	62.83 ± 7.57	62.47 ± 7.43	64.98 ± 7.68	63.02 ± 7.46
	80%TWB	62.49 ± 7.11	65.56 ± 7.39	64.64 ± 7.27	62.79 ± 7.33	65.76 ± 7.35	64.71 ± 7.02	62.93 ± 7.20	65.86 ± 7.34	64.93 ± 7.30
	80%TSE	62.53 ± 7.14	64.77 ± 7.44	63.22 ± 7.31	63.40 ± 7.28	65.08 ± 7.21	63.69 ± 7.43	63.18 ± 7.23	65.25 ± 7.48	63.56 ± 7.43
	Control	62.68 ± 7.14	62.98 ± 7.07	62.67 ± 7.20	62.97 ± 6.97	63.40 ± 7.02	62.95 ± 7.05	63.09 ± 7.09	63.56 ± 7.02	63.08 ± 7.22

SBJ distance (cm)	30%TWB	269.3 ± 9.9	274.8 ± 11.0	269.6 ± 10.0	272.8 ± 9.0	278.0 ± 10.6	274.0 ± 10.5	273.7 ± 10.6	278.3 ± 11.0	273.7 ± 11.1
	80%TWB	273.2 ± 9.6	278.8 ± 11.3	277.4 ± 10.6	271.9 ± 10.9	278.4 ± 11.1	274.4 ± 11.3	271.8 ± 13.1	279.5 ± 14.1	277.6 ± 13.3
	80%TSE	273.9 ± 9.9	277.8 ± 12.4	277.4 ± 10.7	272.3 ± 12.1	278.0 ± 11.3	276.9 ± 10.3	275.0 ± 11.0	274.4 ± 11.9	278.6 ± 13.7
	Control	272.3 ± 10.4	276.6 ± 11.2	274.0 ± 11.5	275.3 ± 9.8	273.3 ± 10.7	274.0 ± 10.5	275.6 ± 10.6	275.0 ± 10.6	275.6 ± 11.3

CCMJ average height (cm)	30%TWB	42.0 ± 3.0	44.9 ± 2.8	42.3 ± 3.0	41.9 ± 2.9	44.8 ± 2.9	42.6 ± 2.9	42.2 ± 2.9	44.6 ± 3.2	42.3 ± 3.4
	80%TWB	42.0 ± 2.9	45.1 ± 3.2	43.7 ± 3.3	41.9 ± 2.9	45.2 ± 3.1	43.7 ± 3.5	42.4 ± 3.3	45.3 ± 3.6	43.9 ± 3.6
	80%TSE	42.7 ± 2.5	44.6 ± 2.8	43.4 ± 3.1	42.6 ± 2.4	44.5 ± 2.9	43.1 ± 3.3	43.1 ± 2.5	44.6 ± 2.7	43.5 ± 3.1
	Control	42.9 ± 1.6	43.5 ± 1.8	43.1 ± 1.9	42.8 ± 1.8	43.3 ± 1.9	43.0 ± 1.8	43.1 ± 1.9	43.2 ± 2.1	43.2 ± 2.0

CCMJ RSImod	30%TWB	0.83 ± 0.12	0.90 ± 0.13	0.84 ± 0.17	0.82 ± 0.13	0.92 ± 0.13	0.85 ± 0.17	0.82 ± 0.14	0.93 ± 0.14	0.86 ± 0.17
	80%TWB	0.84 ± 0.12	0.91 ± 0.12	0.89 ± 0.14	0.84 ± 0.11	0.91 ± 0.13	0.89 ± 0.14	0.84 ± 0.11	0.92 ± 0.13	0.88 ± 0.14
	80%TSE	0.85 ± 0.11	0.92 ± 0.14	0.87 ± 0.13	0.85 ± 0.11	0.93 ± 0.13	0.88 ± 0.12	0.85 ± 0.12	0.93 ± 0.13	0.89 ± 0.12
	Control	0.85 ± 0.10	0.87 ± 0.10	0.85 ± 0.09	0.85 ± 0.10	0.87 ± 0.10	0.85 ± 0.10	0.84 ± 0.11	0.86 ± 0.11	0.85 ± 0.11

**Table:** 

Variable	Intervention	Reliability					

		ICC (90%CL)			CV% (90%CL)		

		0 h	6 h	24 h	0 h	6 h	24 h
CMJ height (cm)	30%TWB	0.99 (0.98 to 0.99)	0.99 (0.98 to 0.99)	0.99 (0.99 to 0.99)	0.62 (0.48 to 0.79)	0.69 (0.56 to 0.84)	0.63 (0.52 to 0.74)
	80%TWB	0.98 (0.97 to 0.99)	0.99 (0.98 to 0.99)	0.99 (0.98 to 0.99)	0.94 (0.77 to 1.12)	0.68 (0.55 to 0.82)	0.80 (0.68 to 0.93)
	80%TSE	0.99 (0.98 to 0.99)	0.99 (0.99 to 0.99)	0.99 (0.98 to 0.99)	0.77 (0.68 to 0.87)	0.70 (0.62 to 0.79)	0.80 (0.68 to 0.93)
	Control	0.99 (0.98 to 0.99)	0.99 (0.98 to 0.99)	0.99 (0.98 to 0.99)	0.75 (0.65 to 0.87)	0.82 (0.71 to 0.94)	0.80 (0.72 to 0.87)

CMJ relative peak power (W/kg−1)	30%TWB	0.98 (0.98 to 0.99)	0.98 (0.98 to 0.99)	0.98 (0.98 to 0.99)	0.96 (0.75 to 1.18)	1.01 (0.78 to 1.23)	0.94 (0.73 to 1.14)
	80%TWB	0.98 (0.98 to 0.99)	0.97 (0.96 to 0.98)	0.98 (0.98 to 0.99)	0.89 (0.69 to 1.08)	1.05 (0.82 to 1.28)	0.81 (0.63 to 0.99)
	80%TSE	0.98 (0.98 to 0.99)	0.98 (0.98 to 0.99)	0.98 (0.98 to 0.99)	1.01 (0.91 to 1.12)	0.95 (0.88 to 1.02)	0.90 (0.75 to 1.07)
	Control	0.98 (0.98 to 0.99)	0.98 (0.98 to 0.99)	0.98 (0.98 to 0.99)	0.91 (0.81 to 1.02)	0.94 (0.83 to 1.06)	1.00 (0.88 to 1.12)

SBJ distance (cm)	30%TWB	0.88 (0.82 to 0.93)	0.89 (0.80 to 0.94)	0.86 (0.77 to 0.92)	1.10 (0.97 to 1.26)	1.13 (0.97 to 1.29)	1.25 (1.04 to 1.47)
	80%TWB	0.77 (0.66 to 0.85)	0.81 (0.68 to 0.90)	0.82 (0.71 to 0.88)	1.74 (1.46 to 2.03)	1.59 (1.31 to 1.89)	1.59 (1.31 to 1.87)
	80%TSE	0.83 (0.74 to 0.90)	0.81 (0.70 to 0.89)	0.81 (0.71 to 0.89)	1.46 (1.26 to 1.67)	1.57 (1.32 to 1.84)	1.64 (1.41 to 1.88)
	Control	0.79 (0.71 to 0.89)	0.81 (0.72 to 0.89)	0.81 (0.70 to 0.89)	1.53 (1.28 to 1.79)	1.63 (1.43 to 1.83)	1.62 (1.42 to 1.84)

CCMJ average height (cm)	30%TWB	0.95 (0.93 to 0.98)	0.95 (0.92 to 0.97)	0.91 (0.86 to 0.95)	1.57 (1.38 to 1.79)	1.30 (1.07 to 1.54)	1.66 (1.30 to 2.12)
	80%TWB	0.89 (0.82 to 0.94)	0.92 (0.88 to 0.96)	0.97 (0.95 to 0.98)	1.88 (1.48 to 2.43)	7.16 (5.87 to 9.25)	7.82 (6.42 to 10.11)
	80%TSE	0.92 (0.86 to 0.95)	0.95 (0.92 to 0.97)	0.88 (0.81 to 0.93)	5.63 (4.62 to 7.28)	6.24 (5.12 to 8.07)	7.00 (5.74 to 9.05)
	Control	0.82 (0.72 to 0.90)	0.86 (0.78 to 0.92)	0.86 (0.78 to 0.92)	3.85 (3.16 to 4.98)	1.48 (1.23 to 1.72)	1.48 (1.22 to 1.73)

CCMJ RSImod	30%TWB	0.93 (0.89 to 0.96)	0.96 (0.94 to 0.98)	0.92 (0.86 to 0.99)	3.66 (2.99 to 4.33)	2.62 (2.20 to 3.04)	4.46 (3.75 to 5.57)
	80%TWB	0.93 (0.89 to 0.96)	0.97 (0.95 to 0.98)	0.96 (0.93 to 0.98)	3.31 (2.84 to 3.78)	2.27 (1.90 to 2.63)	2.90 (2.34 to 3.46)
	80%TSE	0.95 (0.92 to 0.97)	0.96 (0.94 to 0.98)	0.95 (0.92 to 0.97)	2.88 (2.48 to 3.27)	2.61 (2.19 to 3.02)	2.78 (2.33 to 3.23)
	Control	0.96 (0.94 to 0.98)	0.93 (0.89 to 0.96)	0.91 (0.86 to 0.95)	2.61 (2.19 to 3.02)	2.76 (2.28 to 3.23)	3.23 (2.81 to 3.64)

**Table:** 

Variable	Intervention	Sensitivity								

		TE			SWC_0.2_			SWC_0.5_		

		0 h	6 h	24 h	0 h	6 h	24 h	0 h	6 h	24 h

CMJ height (cm)	30%TWB	0.59	0.62	0.44	0.85	0.87	0.89	2.13	2.18	2.22
	80%TWB	0.72	0.56	0.62	0.81	0.85	0.86	2.02	2.13	2.15
	80%TSE	0.56	0.59	0.62	0.75	1.01	0.86	1.88	2.51	2.15
	Control	0.62	0.67	0.58	0.75	0.76	0.71	1.86	1.89	1.78

CMJ relative peak power (W/kg−1)	30%TWB	0.43	0.46	0.42	1.48	1.53	1.48	3.40	3.82	3.71
	80%TWB	0.39	0.49	0.37	1.44	1.47	1.44	3.60	3.67	3.59
	80%TSE	1.03	0.97	0.95	1.43	1.46	1.46	3.57	3.65	3.65
	Control	0.94	0.98	1.04	1.40	1.39	1.41	3.49	3.48	3.54

SBJ distance (cm)	30%TWB	2.53	3.02	3.42	1.97	2.20	2.01	4.93	5.51	5.02
	80%TWB	5.25	5.17	5.08	1.92	2.27	2.13	4.80	5.67	5.32
	80%TSE	4.33	4.74	5.03	1.99	2.48	2.31	4.97	6.20	5.77
	Control	4.50	4.76	4.81	2.05	2.16	2.20	5.12	5.39	5.50

CCMJ average height (cm)	30%TWB	0.63	0.66	0.91	0.58	0.59	0.62	1.46	1.48	1.54
	80%TWB	1.02	0.90	0.63	0.60	0.66	0.68	1.50	1.64	1.71
	80%TSE	0.75	0.63	1.10	0.49	0.56	0.63	1.23	1.40	1.57
	Control	0.74	0.57	0.82	0.35	0.37	0.36	0.87	0.91	0.91

CCMJ RSImod	30%TWB	0.02	0.02	0.03	0.03	0.03	0.03	0.06	0.07	0.08
	80%TWB	0.02	0.02	0.02	0.02	0.03	0.03	0.06	0.06	0.07
	80%TSE	0.02	0.02	0.02	0.02	0.03	0.02	0.06	0.07	0.06
	Control	0.02	0.02	0.02	0.02	0.02	0.02	0.06	0.05	0.05

### Power-oriented single-effort test

CMJ height improved at 6 h post-intervention in 30% 1RM TWB (+6.4 ± 5.0%; *p* < 0.01; d = 1.31), 80% 1RM TWB (+7.4 ± 3.1%; *p* < 0.01; d = 2.49), and 80% 1RM TSE (+3.5 ± 4.2%; *p* < 0.01; d = 0.83) compared with baseline. At 24 h, improvements persisted only in 80% 1RM TWB (+4.8 ± 3.4%; *p* < 0.01; d = 1.45) (Figure 4A). CMJ relative peak power showed a similar trend (Figure 4B).

### Horizontal explosive test

SBJ distance improved at 6 h post-intervention in 30% 1RM TWB (+1.9 ± 1.2%; *p* < 0.01; d = 1.52), 80% 1RM TWB (+2.4 ± 1.6%; *p* < 0.01; d = 1.52), and 80% 1RM TSE (+1.1 ± 1.5%; *p* < 0.01; d = 0.72) compared with baseline. At 24 h, improvement maintained for 80% 1RM TWB (+1.5 ± 1.4%; *p* < 0.01; d = 1.11) and 80% 1RM TSE (+1.4 ± 1.6%; *p* < 0.01; d = 0.91) (Figure 5).

### Power-endurance consecutive-effort test

CCMJ average height increased at 6 h post-intervention in 30% 1RM TWB (+6.7 ± 3.5%; *p* < 0.01; d = 1.96), 80% 1RM TWB (+7.6 ± 6.6%; *p* < 0.01; d = 1.18), and 80% 1RM TSE (+4.2 ± 2.7%; *p* < 0.01; d = 1.53) compared with baseline. At 24 h, improvements persisted only in 80% 1RM TWB (+4.0 ± 3.7%; *p* < 0.01; d = 1.07) (Figure 6A). CCMJ RSImod displayed a similar pattern (Figure 6B).

### Time course of individual response to different priming method from all metrics

Overall responder percentages for all metrics are presented as mean% ± SD%. At 6 h, responder rates for the 30%1RM TWB, 80%1RM TWB and 80%1RM TSE were 84.3 ± 13.1%, 87.7 ± 7.4% and 66.5 ± 14.4%. At 24 h, responder rates were 38.6 ± 14.0%, 73.6 ± 6.6% and 43.9 ± 17.1%. Detailed responder distributions are presented in Figure 7.

## DISCUSSION

This study aimed to determine whether TWB priming exercise elicits greater DPE on explosive performance than TSE. Specifically, we compared low- and high-intensity TWB at 6 h and 24 h, hypothesizing that both would elicit DPE effect. We found that low- and highintensity TWB, as well as high-intensity TSE, significantly improved CMJ height and relative peak power, SBJ distance, CCMJ height, and RSImod at 6 h. However, at 24 h, benefits persisted only in the high-load conditions (80% 1RM TWB and 80% 1RM TSE), while performance returned near baseline in the low-load group (30% 1RM TWB). Additionally, responder rates decreased from 84.3 ± 13.1% to 38.6 ± 14.0% responders across all metrics. (Figure 7) This individual variability underscores the importance of monitoring athlete responses relative to the SWC. Rather than relying solely on group averages, practitioners should identify ‘responders’ to optimize stimulus selection, suggesting that priming strategies may need to be personalized. Overall, this suggests TWB is a promising and effective priming method, with DPE duration influenced more by load than by exercise modality.

### Difference in DPE between TSE and TWB

A key finding was that both low- and high-intensity TWB induced greater improvements in power-oriented single-effort performance (i.e., CMJ height, CMJ relative peak power, and SBJ distance) at 6 h compared with TSE. Responder rates at 6 h were 84.3 ± 13.1% (30%1RM TWB), 87.7 ± 7.4% (80%1RM TWB) and 66.5 ± 14.4% (80%1RM TSE). Moreover, at 24 h, high-intensity TWB outperformed 80% 1RM TSE for several metrics (i.e., CMJ height and relative peak power), as shown by responder rates (78.6% vs. 39.3% and 75% vs. 14.3%, reinforcing TWB as a superior strategy for enhancing explosive performance.

TWB induces greater CMJ performance gains at 6 h and 24 h compared with TSE under the same high-load condition. Moreover, lowload TWB elicited greater improvements across all jump metrics at 6 h than high-intensity TSE, including CMJ height (30% 1RM TWB: 6.4 ± 5.0%, d = 1.31 vs. 80% 1RM TSE: 3.5 ± 4.2%, d = 0.83), CCMJ average height (30% 1RM TWB: 6.7 ± 3.5%, d = 1.96 vs. 80% 1RM TSE: 4.2 ± 2.7%, d = 1.53), and SBJ distance (30% 1RM TWB: 1.9 ± 1.2%, d = 1.52 vs. 80% 1RM TSE: 1.1 ± 1.5%, d = 0.72). These findings suggest that the priming method influences the magnitude of DPE. The spring-assisted rebound in TWB enables participants to execute concentric movements with greater upward concentric velocity and power output during multi-joint actions, making concentric velocity the key factor differentiating TWB from TSE priming exercises. Previous research supports that velocity-oriented, low-intensity priming exercises can elicit DPE. For instance, BT such as loaded and unloaded jumps are both effective as priming exercise in elite male rugby union players. Saez et al. [17]also found that 30% 1RM half-squats did not improve CMJ performance 6 h postexercise, while loaded squat jumps at the same intensity produced significant gains. In the present study, the spring-assisted rebound likely reduced mechanical loading at the start of the upward phase, facilitating faster concentric velocity. Given that movement velocity is a key determinant of explosive performance, the ability to generate force rapidly under an equivalent load provides athletes with a distinct performance advantage [27]. High-velocity training also promotes adaptations such as earlier recruitment of large motor units, higher rate coding, and increased doublet discharge [28] [29]. Therefore, concentric velocity likely plays a central role in driving the DPE.

The TWB exercise features a distinctive bidirectional concentric pattern requiring rapid reciprocal switching, whereby former antagonists become agonists and initial agonists are rapidly inhibited via reciprocal inhibition. Efficient agonist-antagonist coordination, characterized by reduced co-activation, represents an early neuromuscular adaptation to resistance training and contributes substantially to initial strength development [28–30]. Reduced co-activation during contractions reflects neural adaptations that optimize force output [31] [32]. Consistent with this mechanism, the increase in CMJ relative peak power noted in the TWB conditions supports a primarily neural contribution, as early-phase rapid force production is largely driven by neural drive [33]. Therefore, morning-based TWB priming represents a promising strategy for enhancing explosive performance.

### Difference in DPE between 6 h and 24 h

Performance improvements generally declined from 6 h to 24 h, particularly in the 30% 1RM TWB condition, which showed no significant differences from baseline at 24 h, decreasing from 84.3 ± 13.1% to 38.6 ± 14.0% responders across all metrics. Contrastingly, several metrics (i.e., SBJ distance, and CCMJ RSImod) remained significantly elevated in the high-intensity TWB and TSE groups, which suggests that DPE duration depends on priming exercise intensity. These results align with previous research demonstrating that morning-based priming effects peak before 6 h and gradually diminish thereafter [2, 34].

The duration of DPE reflects the balance between physiological stimulus, accumulated fatigue, and subsequent recovery [35]. According to supercompensation theory, priming movement form, intensity, and volume regulate this balance [36]. While higher intensity or volume induces greater fatigue, individuals with sufficient adaptive capacity can recover and assimilate the stimulus, extending the supercompensation phase and sustaining performance enhancement. In this study, participants appeared to tolerate high-intensity priming well, which likely explains why the high-intensity TWB and TSE groups maintained gains across most metrics at 24 h (Figure 7).

Practically, these findings suggest a load-dependent strategy: lowload TWB should be prioritized when the goal is to maximize acute performance within a 6-hour window, as it minimizes residual fatigue. Conversely, high-load TWB appears more effective for sustaining potentiation over a longer duration (up to 24 hours), making it more suitable when the interval between priming and competition is extended.

### Practical applications

Elite-level competitions are often scheduled in the afternoon or evening, which makes morning-based priming exercise a practical strategy to optimize performance. Although DPE can last up to 48 h, performance benefits generally peak within the first 6 h. In this study, all conditions elicited greater improvements at 6 h than at 24 h. Nonetheless, compared with pre-6 h priming, implementing pre-24 h priming before major events (i.e., especially in team sports) is more challenging due to the complex interplay between stimulus and recovery. Consequently, morning-based TWB priming at both low- and high-intensities should be considered an effective and promising DPE strategy for sports demanding high levels of explosive performance. Based on the findings, two practical recommendations emerge:

1) Morning TWB priming is particularly suitable for athletes competing in the afternoon, as it effectively bridges the interval between activation and competition.
2) Low-load TWB should be prioritized when minimizing residual fatigue is essential (e.g., < 6 h), whereas high-load protocols may be more appropriate when longer recovery periods are available (e.g., up to 24 h).

Although the custom spring-assisted device uniquely enables bidirectional concentric actions, the high-velocity nature of the stimulus can be partially replicated in real-world settings. Practitioners may employ low-load agonist-antagonist power complexes to target rapid neural drive and reduced co-activation, which are hypothesized to underpin the observed TWB effects.

## CONCLUSIONS

Morning-based TWB priming exercise, at both low and high intensities, is an effective strategy to achieve DPE 6 h later, with moderate to large improvements, likely to be advantageous for sports requiring explosive performance. High-intensity TWB offers longer-lasting DPE, making it more suitable for complex competition scenarios. This study confirms TWB’s potential to enhance performance and offer new solutions for optimizing explosive performance through targeted priming exercises.

## Data Availability

Data are available from the corresponding author upon reasonable request. The individual depicted in Figure 3 is one of the authors (Yiheng Zeng), who hereby grant full permission for the publication of this image.
